# Dendrimer-conjugated glutaminase inhibitor selectively targets microglial glutaminase in a mouse model of Rett syndrome

**DOI:** 10.7150/thno.41714

**Published:** 2020-04-27

**Authors:** Elizabeth Smith Khoury, Anjali Sharma, Rajasekhar R Ramireddy, Ajit G. Thomas, Jesse Alt, Amanda Fowler, Rana Rais, Takashi Tsukamoto, Mary E. Blue, Barbara Slusher, Sujatha Kannan, Rangaramanujam M. Kannan

**Affiliations:** 1 Department of Anesthesiology and Critical Care Medicine, Johns Hopkins University School of Medicine, Baltimore MD, 21205; 2 Center for Nanomedicine, Department of Ophthalmology, Wilmer Eye Institute Johns Hopkins University School of Medicine, Baltimore MD, 21231; 3 Johns Hopkins Drug Discovery, Johns Hopkins University School of Medicine, Baltimore MD, 21205; 4 Department of Neurology, Johns Hopkins University School of Medicine, Baltimore MD, 21205; 5 Hugo W. Moser Research Institute at Kennedy Krieger Inc., Baltimore MD, 21205; 6 Kennedy Krieger Institute - Johns Hopkins University for Cerebral Palsy Research Excellence, Baltimore, MD 21287; 7 Departments of Chemical and Biomolecular Engineering, and Materials Science and Engineering, Johns Hopkins University, Baltimore MD, 21218

**Keywords:** PAMAM dendrimer, microglia, glutaminase, Rett syndrome

## Abstract

**Background**: Elevated glutamate production and release from glial cells is a common feature of many CNS disorders. Inhibitors of glutaminase (GLS), the enzyme responsible for converting glutamine to glutamate have been developed to target glutamate overproduction. However, many GLS inhibitors have poor aqueous solubility, are unable to cross the blood brain barrier, or demonstrate significant toxicity when given systemically, precluding translation. Enhanced aqueous solubility and systemic therapy targeted to activated glia may address this challenge. Here we examine the impact of microglial-targeted GLS inhibition in a mouse model of Rett syndrome (RTT), a developmental disorder with no viable therapies, manifesting profound central nervous system effects, in which elevated glutamatergic tone, upregulation of microglial GLS, oxidative stress and neuroimmune dysregulation are key features.

**Methods**: To enable this, we conjugated a potent glutaminase inhibitor, *N*-(5-{2-[2-(5-amino-[1,3,4]thiadiazol-2-yl)-ethylsulfanyl]-ethyl}-[1,3,4]thiadiazol-2-yl)-2-phenyl-acetamide (JHU29) to a generation 4 hydroxyl PAMAM dendrimer (D-JHU29). We then examined the effect of D-JHU29 in organotypic slice culture on glutamate release. We also examined GLS activity in microglial and non-microglial cells, and neurobehavioral phenotype after systemic administration of D-JHU29 in a mouse model of RTT.

**Results**: We report successful conjugation of JHU29 to dendrimer resulting in enhanced water solubility compared to free JHU29. D-JHU29 reduced the excessive glutamate release observed in tissue culture slices in a clinically relevant *Mecp2*-knockout (KO) RTT mouse. Microglia isolated from *Mecp2*-KO mice demonstrated upregulation of GLS activity that normalized to wild-type levels following systemic treatment with D-JHU29. Neurobehavioral assessments in D-JHU29 treated *Mecp2*-KO mice revealed selective improvements in mobility.

**Conclusion**: These findings demonstrate that glutaminase inhibitors conjugated to dendrimers are a viable mechanism to selectively inhibit microglial GLS to reduce glutamate production and improve mobility in a mouse model of RTT, with broader implications for selectively targeting this pathway in other neurodegenerative disorders.

## Introduction

Many central nervous system (CNS) diseases and disorders are characterized by increased glutamate production/release and subsequent glutamate excitotoxicity. Microglia and astrocytes play a key role in mediating glutamate production in CNS disorders, with significant impact on neurons and neurobehavior. Glial cell-driven glutamate increases have been shown in CNS disorders characterized by neuroinflammation such as multiple sclerosis, traumatic brain injury, cerebral palsy, HIV-associated dementia and Rett syndrome (RTT) 1-9. In both acute and chronic diseases, elevated glutamate levels often cause a range of problems, varying from excitotoxicity and cell death in acute injury to impaired synaptic plasticity and development as seen in RTT 10,11 a developmental disorder caused by the mutation of the gene responsible for encoding methyl-CpG binding protein 2 (MeCP2), a transcription regulator that has also been shown to be disrupted in autism spectrum disorders 12-14. In the presence of neuroinflammation, a perpetuating cycle exists where elevated glutamate leads to increased pro-inflammatory cytokine levels (e.g. TNFα and IL-1β) 15,16 that in turn can also enhance glutamate production/release 17-19. In the context of neurodevelopment, aberrant glutamatergic signaling can result in abnormal development of the brain and result in life-long impairments in neural function that manifest as motor, cognitive, language, and/or autonomic deficits as is seen in RTT 20-22. Thus, there is a critical need for the development and delivery of drug treatments to decrease glutamate production and release in the CNS.

One approach for reducing glutamate production is inhibition of the enzyme glutaminase (GLS) that is responsible for converting glutamine to glutamate in both neurons and glial cells. Upregulation of GLS expression in the microglia of MeCP2-deficient mice has been implicated in glutamate medicated injury to dendrites and synapses 4. Targeting GLS may be a potentially promising therapeutic strategy in RTT. Although GLS inhibitors show promise for the treatment of certain cancers characterized by GLS upregulation 23-27, historically they have not been suitable candidates for CNS glutamate pathology due to poor solubility and blood brain barrier penetration. Furthermore, since GLS is ubiquitously expressed in many cells throughout the body, systemic administration of GLS inhibitors can have significant systemic side effects 28,29. Taken together, there is a clear need for GLS inhibition in glial cells for the treatment of for complex CNS disorders involving increased glutamatergic transmission such as RTT.

Hydroxyl-terminated poly(amidoamine) (PAMAM-OH) dendrimers provide a viable option to overcome all of these shortcomings including aqueous solubility and brain penetration. Our previous work with PAMAM-OH dendrimers indicated that these nanodevices enhanced the uptake of drugs into the injured brain parenchyma 30-35. PAMAM-OH dendrimers are scalable for easy clinical translation 36 and are capable of crossing an impaired blood brain barrier (as it is in a disease state) and have an innate ability to localize in activated microglia in various animal models including this mouse model of RTT 5,32,37-43. The present study aimed to conjugate a bis-2-(5-phenylacetamido-1,3,4-thiadiazol-2-yl)ethyl sulfide (BPTES) analog *N*-(5-{2-[2-(5-amino-[1,3,4]thiadiazol-2-yl)-ethylsulfanyl]-ethyl}-[1,3,4]thiadiazol-2-yl)-2-phenyl-acetamide (JHU29) 17 to generation 4 (G4) PAMAM-OH dendrimers for the purpose of targeting microglial GLS in a *Mecp2*-knockout (KO) mouse model of RTT. Many BPTES analogs have been created 17,23,44 but JHU29 was chosen as it has greater potency than BPTES and has an amine group as a handle for dendrimer conjugation.

In the following experiments, we demonstrate (1) successful conjugation of JHU29 to G4-PAMAM-OH dendrimer and its physicochemical characterization, (2) improved solubility of JHU29 when attached to PAMAM-OH dendrimer, (3) the ability D-JHU29 to reduce glutamate production *ex vivo* in brain slices harvested from a mouse model of RTT, (4) selective microglial GLS inhibition after systemic D-JHU29 administration, and (5) the impact of systemic D-JHU29 administration on the neurobehavioral deficits seen in this mouse model of RTT.

## Materials and Methods

### Pharmacokinetic assessment of JHU29

Pharmacokinetic studies in mice were conducted according to protocols approved by the Animal Care and Use Committee at Johns Hopkins University. Male CD-1 mice between 25 and 30 g were obtained from Harlan, and maintained on a 12-h light-dark cycle with ad libitum access to food and water. JHU29 was administered to mice as a single intraperitoneal (IP) dose at 10 mg/kg using formulation consisting of 5% DMSO + 2.5% tween + 40% PEG + 52.5% saline v/v. The mice were sacrificed at specified time points post drug administration. For collection of plasma and brain tissue, animals were euthanized with CO_2_, and blood samples were collected in heparinized microtubes by cardiac puncture. Tissues were dissected and immediately flash frozen (-80 °C). Blood samples were spun at 2,000 × *g* for 15 min, plasma was removed and stored at -80 °C until LC/MS analysis.

Prior to extraction, frozen samples were thawed on ice. To quantify JHU29, methanol containing 0.5 µM losartan as an internal standard was added (5 µL/mg to tissue or 5 µL/µL to plasma) in microcentrifuge tubes. Brain tissue was homogenized using a Spex® Geno/Grinder® with stainless steel beads for 1 minute at 1500 RPM. Homogenates and plasma from untreated animals were spiked with JHU 29 from 100 to 0.01 nmol/g or nmol/mL, respectively, by serial dilution to generate standard curves. Tissue and plasma homogenates were vortexed, mixed, and centrifuged (16,000 x g for 5 min at 4°C), supernatants were transferred to a 96 well plate, and 2 µL was injected on an UltiMate 3000 UHPLC coupled to a Q Exactive Focus orbitrap mass spectrometer (Thermo Fisher Scientific Inc., Waltham MA). Samples were separated on an Agilent EclipsePlus C18 RRHD (1.8 µm) 2.1 × 100 mm column. The mobile phase consisted of water + 0.1% formic acid (A), and acetonitrile + 0.1% formic acid (B) at a flow rate of 0.4 mL/min and separation was achieved using a gradient run. Quantification was performed in product-reaction monitoring (PRM) mode using mass transitions of 407.0777>246.0695, 280.0574 (JHU 29) and 423.1695>2073.091, 377.1522 (internal standard).

Pharmacokinetic parameters were analyzed using non-compartmental analysis method as implemented in the computer software program Phoenix^®^ WinNonlin^®^ version 7.0 (Certara USA, Inc., Princeton, NJ). The maximum plasma concentration (C_max_) and time to C_max_ (T_max_) were the observed values. The area under the plasma concentration time curve (AUC) value was calculated to the last quantifiable sample (AUC_last_) by use of the log-linear trapezoidal rule. The brain to plasma ratios were calculated as a ratio of mean AUCs (AUC_0-t,brain_/AUC_0-t,plasma_).

### Synthesis and characterization of intermediates and D-JHU29 conjugate

#### Materials and reagents

JHU29 was synthesized as per a previously published synthesis protocol 23. Reagents included glutaric acid monomethyl ester chloride, (benzotriazol-1-yloxy)tripyrrolidinophosphonium hexafluorophosphate, lithium hydroxide, *N*, *N*-diisopropyl ethyl amine (DIPEA), anhydrous tetrahydrofuran (THF), anhydrous *N*, *N*-dimethylacetamide (DMA) and anhydrous dimethylformamide (DMF) (Sigma Aldrich US) and bifunctional ethylenediamine-core PAMAM dendrimer (OH-D-NH_2_, biomedical grade generation 4 consisting 59 hydroxyl end-groups and 5 terminal amine groups in a solution containing methanol (Dendritech, Midland, MI). To improve the generational purity, the as-received dendrimer methanol solution was evaporated to yield a white solid that was re-dissolved in DI water, transferred to 3000 MWCO dialysis membrane and dialysed against 4 gallon Nanopure water for 36 hours. The solution was stirred during dialysis, and water was changed several times at regular intervals. Purified dendrimer was lyophilized and dried to form a hygroscopic white solid, which was stored at -20^0^C under argon until use. Dialysis membrane (MWCO 3kDa) was purchased from Spectrum Laboratories Inc. All other solvents were used as received in their anhydrous forms. All reactions in the organic medium were performed in standard oven-dried glassware under an inert nitrogen atmosphere. Deuterated solvents dimethylsulfoxide (DMSO-*d6*), methanol (CD_3_OD), water (D_2_O) and chloroform (CDCl_3_) were purchased from Cambridge Isotope Laboratories, Inc.

#### Characterization

*Nuclear Magnetic Resonance (NMR)* spectra were recorded on a Bruker 500MHz spectrometer at ambient temperatures. The chemical shifts in ppm were reported relative to tetramethylsilane as an internal standard for ^1^H NMR spectra. Residual protic solvent of CDCl_3_ (^1^H, δ 7.27 ppm; ^13^C, δ 77.0 ppm (central resonance of the triplet)), D_2_O (^1^H, δ4.79 ppm), and MeOD (^1^H, δ3.31 ppm and ^13^C, δ 49.0 ppm) were used for chemical shifts calibration.

*High performance liquid chromatography (HPLC):* The purity the of D-JHU29 conjugates was analyzed using HPLC (Waters Corporation, Milford, MA) equipped with a 1525 binary pump, 2998 photodiode array (PDA) detector, 2475 multi-wavelength fluorescence detector, and 717 auto-sampler interfaced with Empower software with slight modifications using our previously published methods 45,46. The HPLC chromatograms were monitored at 254 nm and 210 nm using a PDA detector. The mobile phase was water/acetonitrile (0.1% w/w TFA). The column used for this study was a symmetry C18 column (300 A, 5 µM, 4.6 mm x 250 mm) with corresponding guard column. A gradient flow method was used with a flow rate of 1 mL/min and an initial condition of 90:10 (Water/ACN) for 10 minutes, followed by a gradual change to 10:90 ((Water/ACN) for 20 minutes, followed by a gradual change back to the initial condition for 20 minutes.

*Mass spectroscopy:* Accurate mass measurements (HRMS) were performed on BrukermicroTOF-II mass spectrometer using ESI in positive mode and direct flow sample introduction in a CH_3_CN:H_2_O (9:1) solvent system. Either protonated molecular ions [M+nH]^n+^ or adducts [M+nX]^n+^ (X = Na, K, NH4) were used for empirical formula confirmation.

*Size and zeta potential measurements:* The size and zeta potential measurements were measured in triplicates using Zetasizer nano ZS (Marlvern Instrument Ltd. Worchester, U.K.) using our previously reported procedure 47.

*Drug release study:* The release of JHU29 molecules from the D-JHU29 conjugates was determined in PBS (pH 7.4) and citrate buffer (pH 5.5). These conditions were chosen to simulate extracellular physiological (pH 7.4) and internal lysosome (pH 5.5) conditions. D-JHU29 was dissolved in PBS and citrate buffer in two different vials, each at a concentration of 3mg/mL; and both solutions were kept on a shaker at 37^o^C. From each solution, 200 µL of sample was collected at various time-points and was diluted by adding 200 µl of methanol. The samples were stored at -80^o^C and were later analyzed using HPLC. The area under the curve for the free drug peak in the sample was recorded from HPLC that was then converted to the amount of free drug released by correlating with standard HPLC calibration curve of the free drug with known concentrations.

#### Synthesis

*Synthesis of compound 3:* DIPEA (26µL, 0.145 mmoles) was added to a stirring solution of JHU29 (compound** 1**, 25mg, 0.058 mmoles) in a mixture of 1:1 DCM/DMA (10mL), and the solution was stirred for 5 minutes under inert atmosphere. Glutaric acid monomethyl ester chloride (compound** 2**, 13.6mg, 0.081 mmoles) was added to the reaction flask and the stirring was continued for 24 hours. Upon completion, the solvents were evaporated and the crude fraction was purified using column chromatography to afford compound **3** as pure product.

*^1^H NMR* (500 MHz, DMSO): δ 12.68 (s, 1H), 12.40 (s, 1H), 7.43 - 7.16 (m, 5H), 3.81 (s, 2H), 3.59 (s, 3H), 3.26 (td, J = 7.1, 2.6 Hz, 4H), 2.94 (t, J = 6.9 Hz, 4H), 2.51 (t, 2H), 2.36 (t, J = 7.4 Hz, 2H), 1.85 (p, J = 7.4 Hz, 2H). **Figure S1**

*Mass* (ESI): m/z Theoretical: 533.12 Obtained: 535.13 (M+2)^+^ **Figure S2**

*Synthesis of compound 4:* A solution of LiOH (13.5mg, 0.561 mmoles) dissolved in water (1mL) was slowly added to the stirring solution of compound** 3** (50mg, 0.093 mmoles) in 1:1 (THF/water) 5mL. The reaction mixture was stirred at room temperature for 24 hours. Upon completion, the reaction mixture was acidified with 1N HCl and the product was extracted with DCM. The organic layer was dried over sodium sulfate and evaporated to afford the compound **4** as product.

*^1^H NMR* (500 MHz, DMSO): δ 12.73 (s, 1H), 12.44 (s, 1H), 12.16 (s, 1H), 7.52 - 7.22 (m, 5H), 3.85 (s, 2H), 3.31 (td, J = 7.1, 3.5 Hz, 4H), 2.99 (t, J = 7.1 Hz, 4H), 2.55 (t, 2H), 2.31 (t, J = 7.3 Hz, 2H), 1.93 - 1.75 (m, 2H). **Figure S3**

*Mass* (ESI): m/z Theoretical: 519.11 Obtained: 521.11 (M+2)^+^ **Figure S4**

*Synthesis of compound 6:* PyBOP (29.14mg, 0.056 mmoles) and DIPEA (10µL) was added to a stirring solution of bifunctional dendrimer (compound **5**, 38mg, 0.002 mmoles) and compound **4** (15mg, 0.028 mmoles) in anhydrous DMF, at ^o^0C under inert atmosphere. The reaction mixture was stirred at room temperature for 48 hours. Upon completion, the reaction mixture was diluted with DMF and dialyzed against DMF for 12 hours followed by water dialysis for 24 hours. The dialysis solvents were changed every 3 hours. The aqueous solution was then lyophilized to afford D-JHU29 (compound **6**) as pure product.

^1^H NMR (500 MHz, DMSO): δ 12.68 (s, 5H, JHU-29 amide *H*), 12.37 (s, 5H, JHU-29 amide *H*), 8.26 - 7.57 (m, 124H, dendrimer-amide *H*), 7.42 - 7.15 (m, 5H, JHu-29 Ar *H*), 4.04 (bs, 10H, ester *H*), 3.81 (s, 10H, JHU-29 Ar CH_2_*H*), 3.57 - 3.23 (m, JHu-29 and dendrimer -C*H*_2_), 3.21 - 2.99 (m, dendrimer -C*H*_2_), 2.97 - 2.82 (m, JHU29 and dendrimer -C*H*_2_), 2.67 (m, JHU-29 and dendrimer -C*H*_2_), 2.43 (m, dendrimer -C*H*_2_), 2.36 - 1.97 (m, dendrimer -C*H*_2_), 1.72 - 1.50 (m, JHU-29-C*H*_2_). **Figure S5**

### Evaluation of D-JHU29 efficacy

***Ex vivo hippocampal slice culture.*** A slice culture protocol was conducted as previously optimized for working with fragile tissue 35. Briefly, coronal slices (300 μm thick) containing the hippocampi were cut from fresh brain tissue of both WT and *Mecp2-*KO mice (5 weeks of age) and placed individually on a tissue culture insert to which 0.3 mL of media (Hank's Basic Salt Solution without calcium and magnesium + 0.64% glucose and 1% penicillin) was added (Figure 4A). After incubating 24 hours at 37**°**C, media was removed and the treatment media (2:1 Minimum Essential Media:HBSS without calcium and magnesium + 1% penicillin) with JHU-29 or D-JHU-29 was applied to the slices. Dendrimer conjugated-JHU29 was given at the same drug concentrations as free JHU29 (100 μg/ml). Twenty-four hours later, media were collected and snap frozen (**Figure 4A**). All experimental conditions had n = 3.

***Glutamate Assay.*** Extracellular glutamate levels were quantified in the collected media samples using Amplex Red Glutamic Acid Assay (Invitrogen). Reaction solution was prepared as per manufacturer's instructions. Fifty μl sample and 50 μl reaction mix were pipetted into a 96 well plate, incubated for 30 minutes and then read in a plate reader at absorbance/emittance of 530/590nm. Concentrations were normalized to tissue slice mass to account for inherent volumetric decreases in *Mecp2*-KO brain 48.

***Subjects.**** Mecp2^+/-^*(Mecp2*^tm1.1Bird^*/J) and CX3CR1^GFP/GFP^ (Cx3cr1^tm1Litt^/J) mice were initially acquired from Jackson Laboratory (Bar Harbor, Maine). *Mecp2* is found on the x chromosome, thus breeding *Mecp2^+/-^*female heterozygous mice with wild type (WT) mice yielded *Mecp2-*KO males, *Mecp2-*heterozygous females, and WT males and females. Breeding *Mecp2^+/-^*female heterozygous mice and CX3CR1^GFP/GFP^ male mice resulted in the same *Mecp2* genotypes with all mice being CX3CR1^GFP/+^. CX3CR1-GFP heterozygous mice were used to minimize any functional alteration that might occur from GFP insertion. WT mice were used for pharmacokinetic analyses. All mice were housed in a vivarium maintained at 72**°**F and 40% humidity with a light cycle of 14 hours on: 10 hours off with lights on at 7AM. All mouse pups were weaned at 28 days of age. No more than 5 mice were housed together per cage. Mice had free access to food and water for the duration of the experiment. All housing and experimental procedures were in accordance with ARRIVE guidelines and approved by the Johns Hopkins Animal Care and Use Committee.

***Formulations preparation for in vivo studies.***The formulations were prepared on a free-drug equimolar basis for both JHU29 and D-JHU29. D-JHU29 formulations were prepared by dissolving the required amount of conjugate in 0.9% sterile saline following filtration through 0.2µm sterile filters (Pall corporation). Free JHU29 formulation was prepared in 5% DMSO + 2.5% tween + 40% PEG 400+ 52.5% sterile Saline.

***Fluorescent activated cell sorting (FACS) and glutaminase inhibition assay.**** Mecp2-KO* CX3CR1^GFP/+^ mice (5-7 weeks of age) were injected with D-JHU29 or saline twice-72 hours apart (n = 3-5 per group). Twenty-four hours after the last injection, mice were euthanized, perfused with saline, and brains processed to create a single cell suspension. For FACS, brains were minced on ice and incubated in StemPro Accutase (ThermoFisher Scientific) for 30 min. Cell pellets were resuspended in Dulbecco's Phosphate Buffered Saline (Corning Cellgro). Suspensions were triturated and the supernatants passed through a 70 μm filter. After that, cells were spun down and supernatant aspirated. Cells then were subjected to a debris removal process (Debris removal solution; Miltenyi Biotec). After debris was removed, cells were resuspended in flow cytometry buffer (eBioscience). Cells were sorted using FACSAria flow cytometer (BD Biosciences; see **Fig S8** for gating scheme). GFP+ and GFP- cells were pelleted and flash frozen on dry ice. Glutaminase activity was measured by the ability of the enzyme in the sample to convert [^3^H]-glutamine to [^3^H]-glutamate. Both CX3CR1+ cells (GFP+) and CX3CR1- (GFP-) cells were collected for the analysis. Cell lysates were exposed to [^3^H]-glutamine (0.09 µM, 2.73 µCi) for 180 min at RT. The assay was terminated upon the addition of imidazole buffer (20 mM, pH 7). [^3^H]-Glutamate, the reaction product, was then eluted with 0.1 N HCl and analyzed for radioactivity using Perkin Elmer's TopCount instrument. Finally, total protein measurements were taken (BioRad's Detergent Compatible Protein Assay kit) and data presented as fmol/mg/h.

***Neurobehavioral Evaluation.*** At 2 weeks of age,* Mecp2*-KO and WT mice (n = 6 per group) began twice-weekly (Mon/Fri) intraperitoneal (IP) injections of saline (WT, *Mecp2-*KO), or D-JHU29 at 10 mg/kg on a drug basis (*Mecp2-*KO). Starting at 5 weeks of age and continuing through 7 weeks of age, mice underwent behavioral testing including open field, rotarod, novel object recognition and full-body plethysmography. Once testing was complete (8 weeks of age), mice were euthanized with an overdose of pentobarbital (Euthasol, Virbac Animal Health). Similar to previous publications by our group and others, we scored classic phenotypic features on a scale of 0-3 (0- not present; 3 - constant/severe). These features included mobility, gait, respiration, and paw clench (see appendix for scoring rubric). All features were scored and then added together to form a composite neurobehavior score. With five sub-scores, the highest combined score was 15. A higher score indicated a more severe overall phenotype. Rotarod testing was conducted with one day of training and another day of testing. On the training day, mice were trained to walk on a rotarod at a fixed speed of 4 rpm. After two successful trials of 2 minutes each, mice were considered trained. The next day (test day) the mice were placed on the rotarod for five trials in which the rod started at 4 rpm and accelerated at 0.3rpm/s. Mice were given two minutes between each trial. Latency to fall from the rotarod was recorded for each trial and averaged across all trials. For open field, mice were placed in an enclosure (38 x 26.5 cm) for 7 minutes (2 min acclimation, 5 min trial). Using Noldus Ethovision scoring software (version XT 11, Noldus), mice were taped from overhead and measures of distance traveled and velocity were obtained. After open field testing, mice were placed back into the enclosure with two identical objects for five minutes. Interaction time was calculated for both objects. One hour later, one of the objects was replaced and the mice were placed back into the enclosure for another five minutes and allowed to investigate both objects. Time spent interacting with both the novel and the familiar object were tabulated and the percentage of investigation time spent with the novel object was calculated. Plethysmography was conducted as described by Glaab et al. 49. In brief, a sealed cylinder with a small air vent was connected to an air pressure sensor. This cylinder was then connected to a pressure transducer for input into LabChart Software (AD instruments). The mice were placed in the cylinder for 15 minutes (5 min habituation, 10 min test). Quiet/still moments in which the mice were resting and not moving or grooming were noted in the trace and spliced out for calculations. Using MATLAB, breath rate was calculated from the pressure trace.

***Statistics.*** All statistics were conducted using Graphpad Prism version 7. All experiments with more than two levels of the independent variables were analyzed using one-way ANOVAs. When appropriate, Dunnett multiple comparisons were used for post-hoc analyses. All tests were two-tailed with a *p* value threshold of 0.05.

## Results and Discussion

### Free JHU29 has poor brain penetration

**Figure 1:**
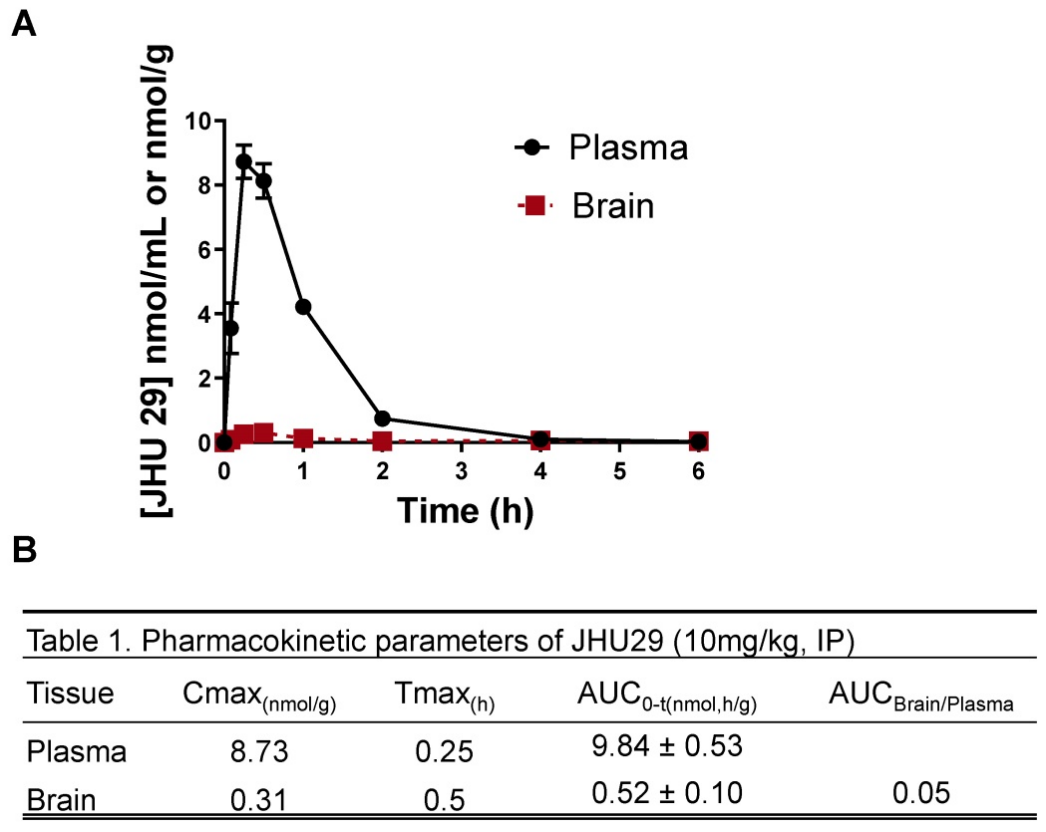
** (A)** Pharmacokinetics of JHU29 in mouse plasma and brain. JHU 29 was dosed at 10 mg/kg intraperitoneally and plasma and brain were collected at various timepoints 0-6hrs. (B) Pharmacokinetic parameters of JHU29.

Wild-type mice were administered JHU29 (10mg/kg IP) and plasma and brain tissue was collected at various time points post-administration to acquire pharmacokinetic data. Following IP administration JHU29 exhibited plasma C_max_ of 8.73 and AUC_0-t_ of 9.83 ± 0.53. In brain low exposures were observed with C_max_ of 0.31 and AUC0-t of 0.52 ± 0.10, giving a poor brain-to-plasma AUC ratio of 0.05 (**Figure 1**). Moreover, considering that cerebral blood volume generally accounts for 4% of total blood volume, it is likely that the 5% of drug seen in the brain is largely accounted for by drug present in the cerebral blood. These data suggest that JHU29 has negligible brain penetration and thus in need of a better mechanism for delivery to brain targets.

### Synthesis, purification and characterization of dendrimer-GLS inhibitor conjugate (D-JHU29)

**Figure 2:**
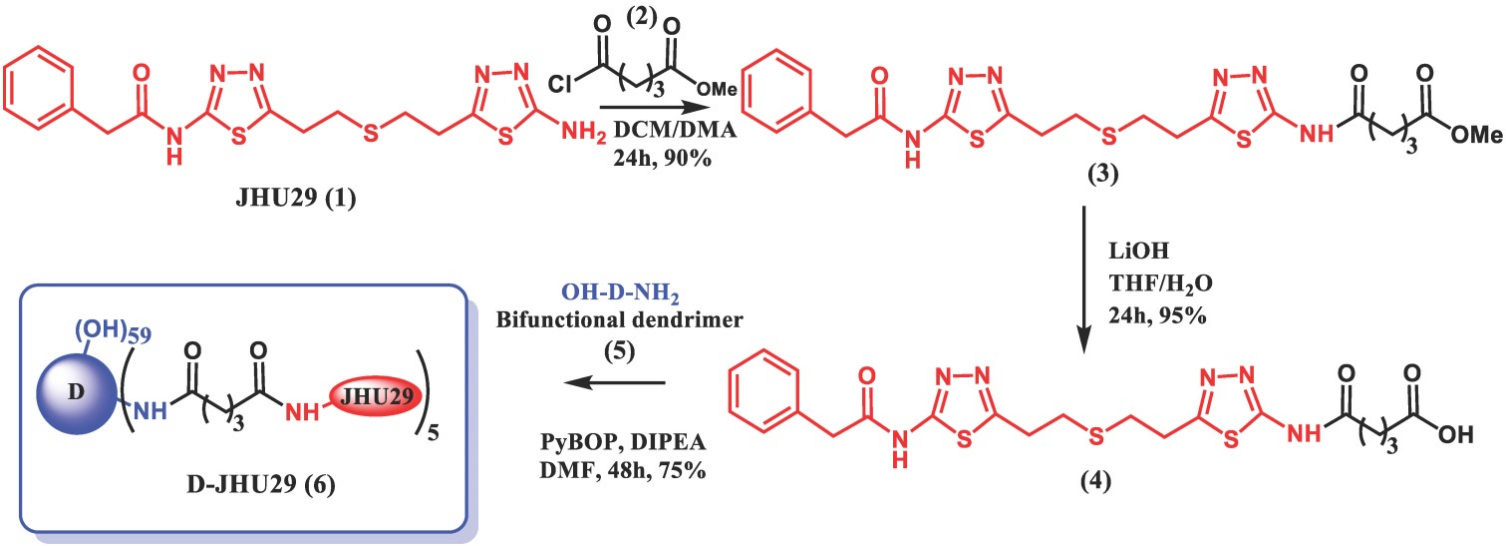
Schematic representation of synthesis of D-JHU29.

**Figure 3:**
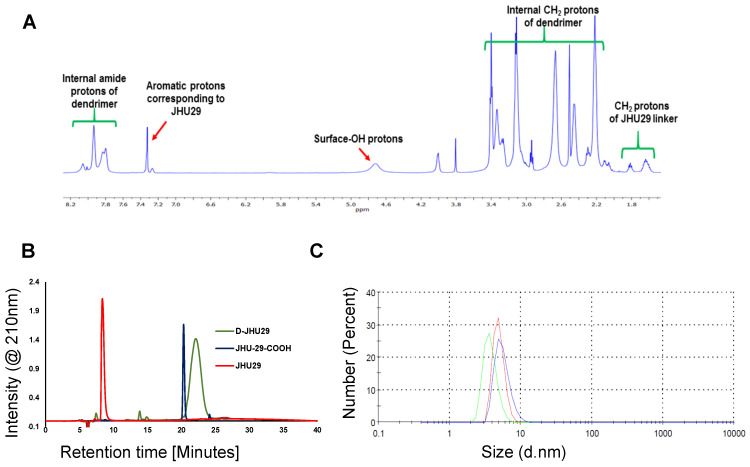
** A.** ^1^H NMR spectrum of D-JHU29 showing the peaks from dendrimer protons and JHU29 protons confirming the conjugation**; B.** Comparative HPLC chromatogram of the free drug **(1)**, JHU29-COOH linker** (4)** and D-JHU29** (6)**. **C.** Hydrodynamic diameter as measured by the dynamic light scattering (4.8 ± 0.9).

To enhance the aqueous solubility and brain penetration of JHU29, we conjugated it to the surface of hydroxyl PAMAM dendrimers. The synthesis of D-JHU29 dendrimer conjugate was carried out in three steps. In the first step, JHU29 (compound **1**, **Figure 2**) was modified to attach a linker utilizing its amine terminal group by reacting with glutaric acid monomethyl ester chloride (compound **2**). The resulting compound **3** was obtained as a methyl ester derivative. The structure of the compound **3** was confirmed by ^1^H NMR and mass spectroscopy (**Figures S1 and S2**). ^1^H NMR showed peaks corresponding to the methylene protons of the linker (δ 1.5 to 3.5 ppm) and the methyl ester protons at δ 3.59 ppm. The methyl ester derivative **3** was further hydrolyzed using mild basic conditions in the presence of lithium hydroxide to obtain a free carboxylic acid group on the drug linker (**4**). The ^1^H NMR clearly revealed the disappearance of methyl protons at 3.59 ppm (**Figure S3**). The purpose of the attachment of the linker was to modify the drug to have a reactive handle that could participate in the reaction with the groups on the dendrimer surface. The free carboxylic acid group on the compound **4** was then reacted with bifunctional dendrimer (**5**) containing 5 amine groups and 59 hydroxyl surface groups using PyBOP and DIPEA. The crude compound was purified by dialysis to make the D-JHU29 conjugate. ^1^H NMR was used to analyze the structure of the final conjugate and the number of drug molecules attached per dendrimer (**Figure 3A**). The comparison of integration of internal amide protons to the aromatic protons of JHU29 revealed the attachment of 5 molecules of JHU29 per dendrimer on an average, suggesting a drug loading of 12% by weight. All the compounds were characterized using ^1^H NMR and mass spectroscopy (**Figures S1-S6**). The comparative HPLC chromatograms of the JHU-29 (**1**), JHU29-COOH linker (**4**) and the final D-JHU29 conjugate (**6**), showed a clear shift in the retention time at each step with the conjugate showing a higher peak retention time (~22 min; >95% purity) compared to free drug (14.5 min) and the drug linker (~20 min), further confirming the successful conjugation (**Figure 3B**). The hydrodynamic diameter of D-JHU29 was 4.8 ±0.9 nm as analyzed by dynamic light scattering (**Figure 3C**). D-JHU29 exhibited a nearly neutral (+3.5±2 mV) zeta potential (**Figure S7**). Small size and a neutral zeta potential is a key requirement for dendrimer conjugates to move freely in the brain parenchyma and target activated microglia and macrophages 50.

### *In vitro* drug release study

**Figure 4:**
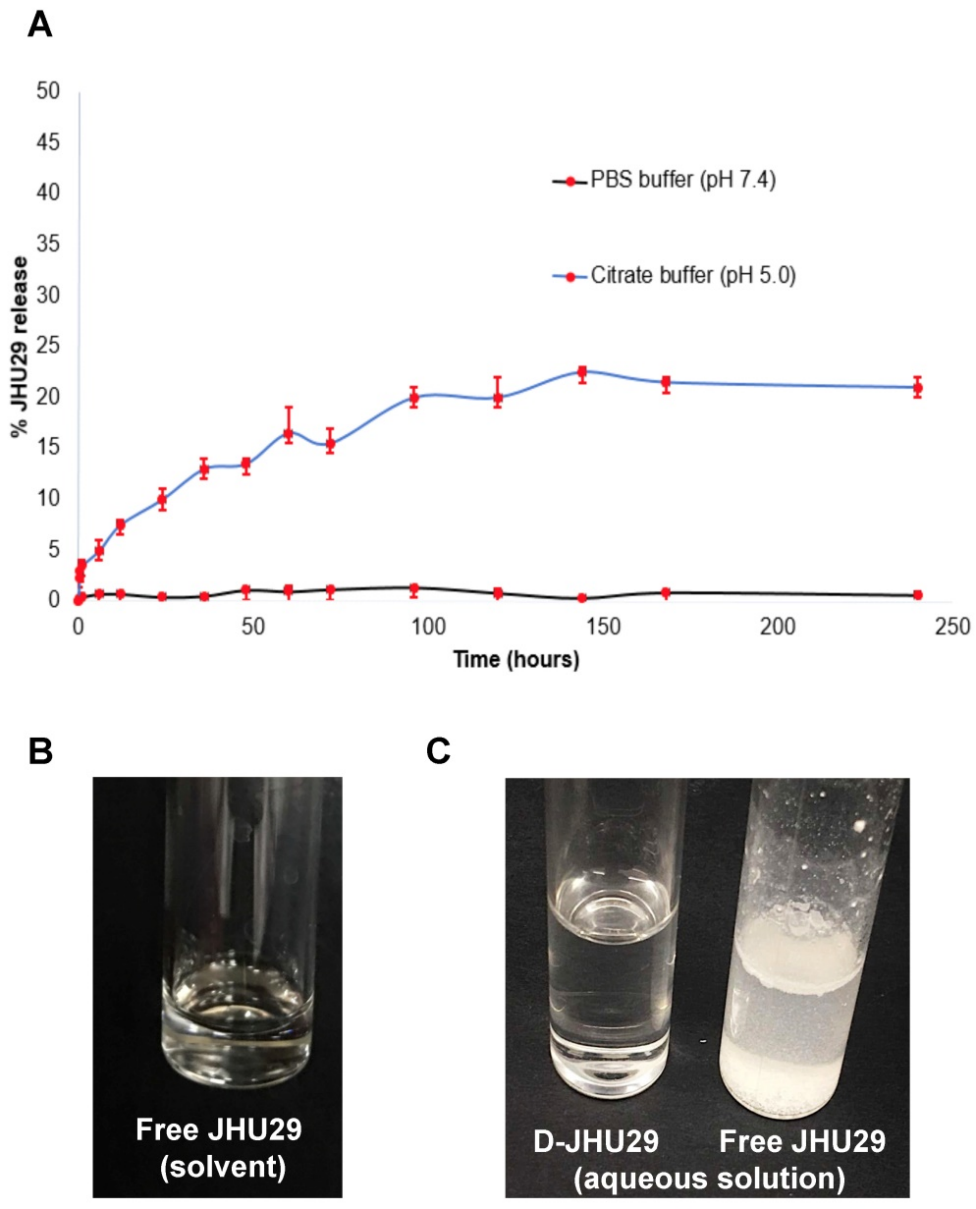
** A.** *In vitro* drug release profile of D-JHU29 at physiological pH (7.4; black line) and lysosomal pH (5.0; blue line). **B.** Solubility of free JHU29 in 5%DMSO + 2.5% tween + 40% PEG400 + 52.5% sterile saline at a concentration of 5mg/mL. **C.** Comparison of aqueous solubility of free JHU29 to D-JHU29 at 1mg/mL on free JHU29 basis.

The stability and release of JHU29 molecules from the D-JHU29 conjugate was determined in PBS (pH 7.4) and citrate buffer (pH 5.5) (**Figure 4A**). These conditions were chosen to simulate the extracellular physiological pH (pH 7.4) and internal lysosome (pH 5.5) conditions 39,51. The percent of released free drug from D-JHU29 was quantified using HPLC via a standard calibration curve. D-JHU29 conjugate was highly stable at physiological pH 7.4 with <2% free drug release over 10 days. At a pH of 5.5, ~25% of the drug was released over 7 days (**Figure 4A**). In this case, release of the drug was not necessary because the active site of JHU29 was still available to bind to the target enzyme.

### Dendrimer conjugation improves solubility of JHU29

Due to the poor water solubility of free JHU29, it requires a complex vehicle solution (5% DMSO + 2.5% tween + 40% PEG + 52.5% Saline) for systemic injections (**Figure 4B**). Conjugation with the highly water-soluble PAMAM dendrimer has been a successful technique to enhance the aqueous solubility of free drugs 52. In order to maintain the aqueous solubility and hydrophobic/hydrophilic balance of the final D-JHU29 conjugate, we only attached 5 molecules of JHU29 on average per dendrimer, which resulted in a conjugate with ~100-fold increase in the water solubility of JHU29. While free JHU29 has a water solubility <10µg/mL, D-JHU29 demonstrated the enhanced solubility as 1mg/mL on free JHU29 basis (**Figure 4C**).

### D-JHU29 treatment of *Mecp2*-KO organotypic slice culture results in decreased glutamate release

**Figure 5:**
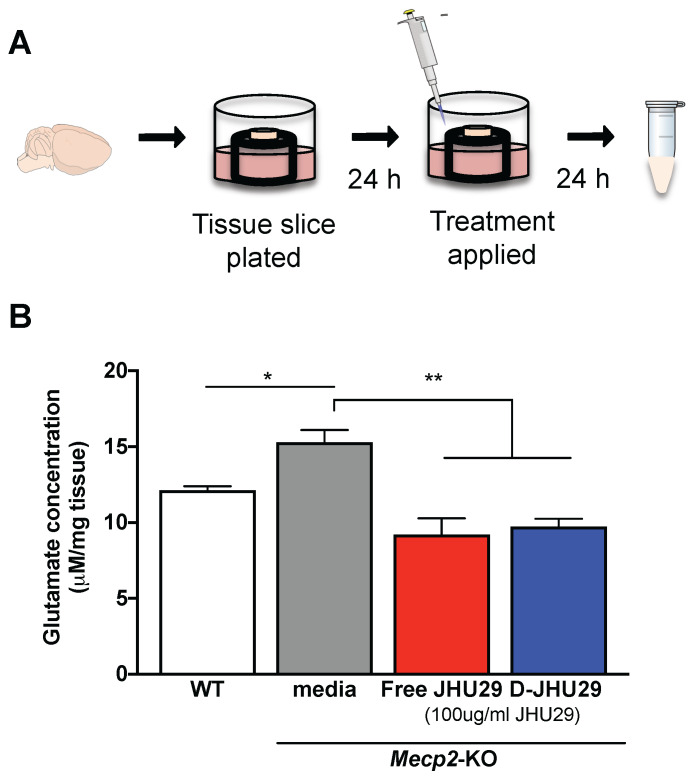
** D-JHU29 decreased the abnormal elevated glutamate release in brain slices from *Mecp2*-KO mice. A.** Coronal slices (300 μm thick) containing the hippocampi were cut from fresh brain tissue of both WT and *Mecp2-*KO mice; each slice was plated individually. Twenty-four hours after plating, the culture media was changed to media alone, media +JHU29 or media + D-JHU29. Media was collected 24 hours later and analyzed for glutamate concentration.** B.** *Mecp2*-KO hippocampal slice cultures from 5-6 week old mice show increased glutamate production in tissue culture. D-JHU29 and free JHU29 (both at 100 μg/ml JHU29) decreased extracellular glutamate levels in *Mecp2*-KO slice culture. * *p* < 0.05, ** *p* < 0.01

Treatment with 100µg/ml of JHU29 delivered via dendrimer or as free drug reduced extracellular glutamate release in hippocampal tissue slices from *Mecp2*-KO mice (**Figure 5B**), demonstrating that dendrimer conjugation did not alter drug efficacy. Previous studies showing increases in glutamate levels in cortical and hippocampal regions of MeCP2-deficient mice and in CSF of patients 53-55 indicate that overproduction of glutamate may play a significant role in the pathology in RTT. Increased neuronal excitability, decreased astrocytic clearance of glutamate, and increased production/release of glutamate by microglia may all contribute to increased extracellular glutamate 4,54,56. This increase in glutamate has been shown in mouse models to be functionally related to sleep disturbances, seizures, cognitive dysfunction, and other aspects of the neurobehavioral phenotype commonly seen in both patients and mouse models of RTT 55,57-61. Thus, targeting this aspect of neuropathology could play a crucial role in the remediation of these aspects of the phenotype.

### D-JHU29 targets *in vivo* microglial glutaminase activity

**Figure 6:**
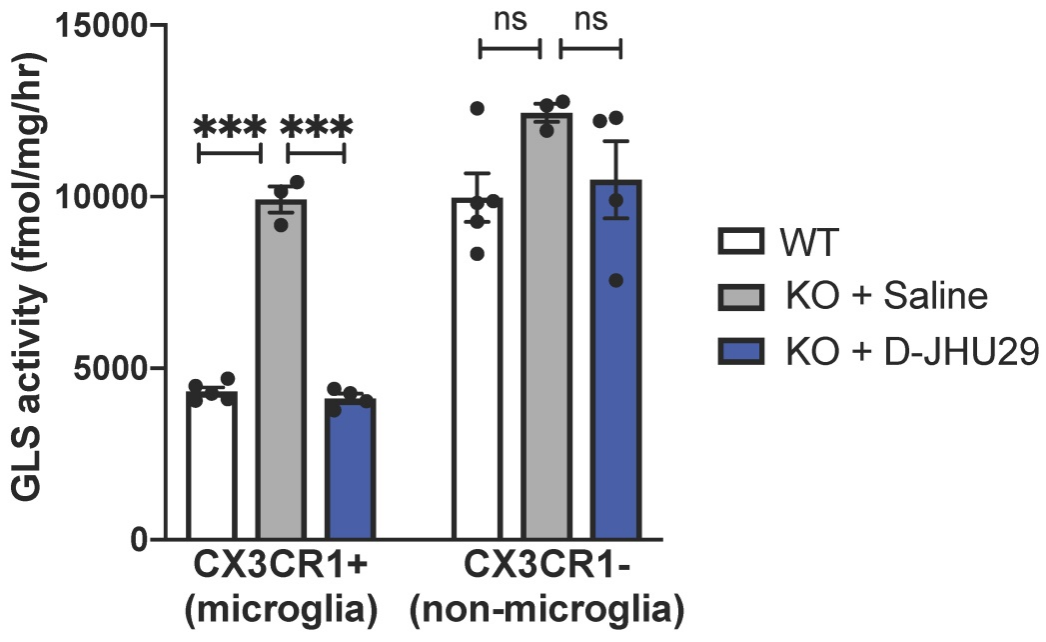
***In vivo* glutaminase (GLS) activity.** WT and *Mecp2*-KO expressing CX3CR1^GFP+^ were injected intraperitoneally with saline or D-JHU29 (10mg/kg on JHU29 basis). Upon sacrifice, Cx3CR1^+^ cells labeled with GFP (microglia) and CX3CR1^-^ cells (GFP-, non-microglia) cells were collected. GLS activity was significantly higher in *Mecp2*-KO CX3CR1^+^ cells than compared to WT CX3CR1^+^ cells and significantly reduced in *Mecp2*-KO CX3CR1 cells after D-JHU29 treatment. Further, no significant effect of D-JHU29 was seen in the CX3CR1^-^ cells, demonstrating a specific effect of D-JHU29 in microglia. *** p < 0.001

We have previously shown that hydroxyl PAMAM dendrimers (without a need for targeting ligands) preferentially localize in activated glia upon systemic administration in multiple small and large animal models of neuroinflammation irrespective of the attachment of therapeutic agents 5,31,62-65,32-34,38,39,43,45,46. Further, we find that peak uptake of G4 PAMAM-OH is reached at 1 hour and sustained through 24 hours 46. The hydroxyl dendrimer used here has also been shown to target activated microglia in this mouse model of RTT within 24 hours of administration 43. To demonstrate the specific activity of D-JHU29 on microglial glutaminase, *Mecp2-KO* CX3CR1^GFP/+^ mice (5-7 weeks of age) were injected with D-JHU29 or saline twice-72 hours apart. Mice were euthanized 24 hours after the second injection and CX3CR1^GFP/+^ cells (microglia) and GFP negative cells (non-microglia) were collected via fluorescent-activated cell sorting (FACS) and GLS activity was measured. Phenotype-expressing mice 5-7 weeks of age showed pathologically high microglial GLS activity compared to litter-matched saline treated WT mice in CX3CR1^GFP^ microglia. Further, this up-regulation in microglial GLS activity was normalized (e.g. decreased) by IP injection of D-JHU29 (10 mg/kg on a JHU29 basis) to WT levels (**Figure 6**). This change in GLS activity was not observed in the non-microglial cell fraction. While the non-microglial cells from the *Mecp2-KO* CX3CR1^GFP/+^ mice demonstrated a non-significant trend of increase in GLS activity versus WT mice, there was no significant change with D-JHU29 treatment (**Figure 6**). These data demonstrate that dendrimer conjugation using G4-PAMAM-OH dendrimers is (1) an effective means of systemically delivering a drug to affected areas of the brain and (2) an effective way to target microglial-specific mechanisms.

### Targeting glial-based glutamate dysfunction via GLS inhibition improves select behavioral dysfunctions in *Mecp2-KO* mice

**Figure 7:**
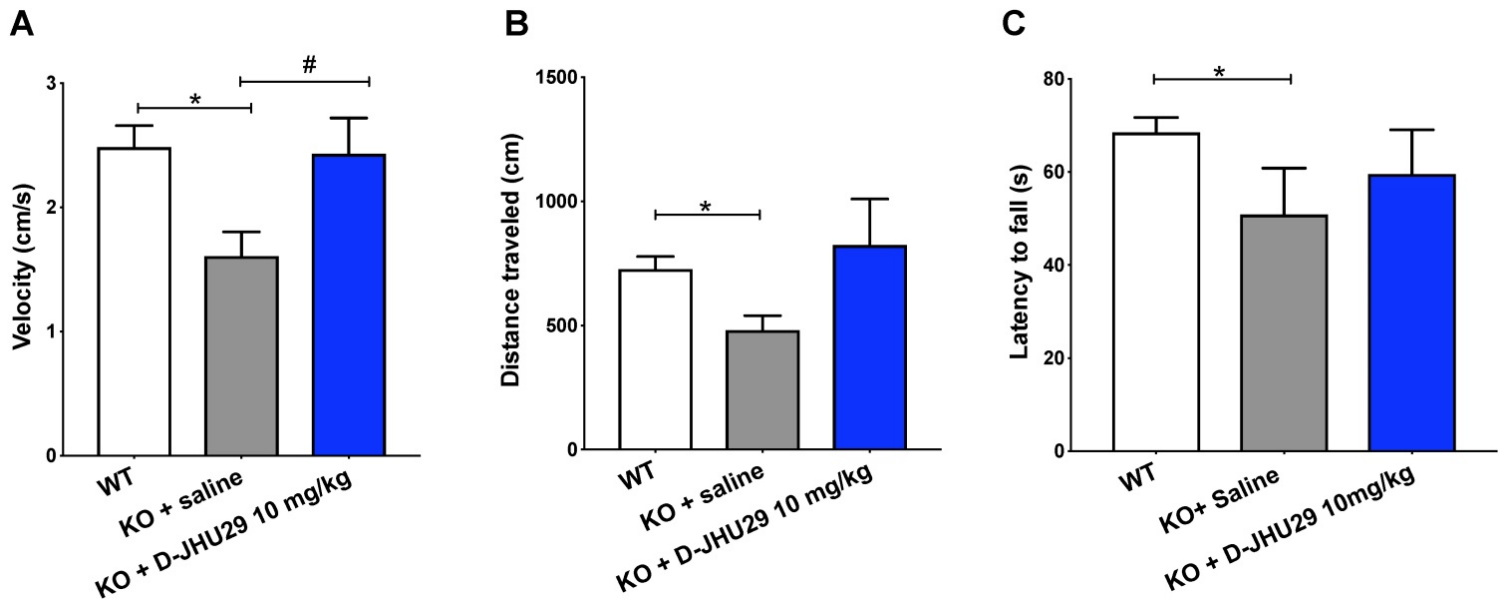
** Motor assessments in *Mecp2*-KO mice after twice weekly D-JHU29 treatment. A.** Velocity of open field movement at 5 weeks of age was modestly improved with D-JHU29 administration (10 mg/kg on a JHU29 basis).** B.** Distance in the open field also showed a trend of improvement with D-JHU29 10 mg/kg administration. **C.** No improvement in rotarod was observed with D-JHU29 treatment. # p = 0.07, *p < 0.05, ** p < 0.01

Research aimed at understanding the pathophysiology and progression of RTT has uncovered an important role of aberrant glutamatergic processes in the production of malfunctioning neural networks and behavioral phenotype. To better understand the role of microglial GLS inhibition in RTT, we evaluated the impact of D-JHU29 administration in a mouse model. *Mecp2*-KO mice were injected twice weekly with D-JHU29 (*10 mg/kg IP* on a JHU29 basis) or saline beginning at postnatal day (PD) 14, the time of behavioral phenotype onset in this model. Mice were tested beginning at 5 weeks of age (PD 35) for mobility and motor function with the open field and rotarod tests. Modest improvements in the velocity of movement were observed with D-JHU29 treatment (**Figure 7A**). However no significant improvements in distance traveled or in accelerating rotarod performance were observed in D-JHU29-treated mice as compared to saline-treated *Mecp2-*KO mice (**Figure 7B, 7C**). This indicates that mobility but not skilled motor behavior may be improved as a consequence of D-JHU29 administration.

Non-motor aspects of the RTT phenotype were also assessed including a neurobehavioral score that is analogous to a clinical severity score used in RTT patients, which assessed paw clenching behavior as a measure of stereotypic behavior. Respiratory rate and spatial memory (novel object recognition) were also assessed in these mice as they have previously been shown to be disrupted and related to changes in glutamate 60,66,67. There was no difference between vehicle- and D-JHU29 treated animals in overall neurobehavioral score, paw clench score, respiratory function and learning (tested by novel object placement) (**Figure S9**).

Preclinical work suggests that intervening in glutamate-based neuropathologies can improve behavioral outcomes in MeCP2-deficient mice and have even led to the investigation of therapies such as dextromethorphan and ketamine in patients 68,69. Specifically, mice treated with the NMDA receptor antagonist ketamine showed improvement in paw clasping, latency to fall off the rotarod, respiratory function and neural circuit functionality 70-72. However, ketamine has other downstream cellular mechanisms of action such as increasing BDNF levels and mTOR activation, apart from NMDAR antagonism, which also could have neuroprotective effects 70,73. The findings in this study support the hypothesis that although targeted reduction of glutamate production by microglia is neuroprotective, it is not adequate for the treatment of such a complex developmental disorder such as RTT. However, this mechanism of specific inhibition of glutamate by activated microglia using D-JHU29 either alone or in combination with other therapies may be effective in acute and chronic conditions where neuroinflammation and glutamate excitotoxicity play a role in the injury. Future work will evaluate the efficacy of D-JHU29 in combination with other drugs for the treatment of RTT.

## Conclusion

Targeted attenuation of a specific disease mechanism (e.g. GLS production) in activated microglia opens new avenues for understanding disease mechanisms and developing therapies for CNS disorders. Here we demonstrate that the hydroxyl PAMAM dendrimer (without a need for targeting ligands) is an effective system to improve aqueous solubility of BPTES analogs and delivery them to 'dysregulated' microglia in a mouse model of RTT. By attaching such an inhibitor to a G4-PAMAM-OH dendrimer, we showed that we can eliminate the use of non-saline solvents for formulation purposes as well as (1) decrease glutamate *ex vivo,* (2) specifically inhibit microglial GLS activity *in vivo*, and (3) improved motor function in a mouse model of RTT. This dendrimer delivery strategy not only has future therapeutic relevance for RTT and other diseases marked by glial-based pathologies, but also is valuable for elucidating a mechanistic understanding of drug action in glia and downstream functional consequences.

## Supplementary Material

Supplementary figures and information.

## Abbreviations

BDNF: brain derived neurotrophic factor
BPTES: Bis-2-(5-phenylacetamido-1,3,4-thiadiazol-2-yl)ethyl sulfide
FACS: fluorescent activated cell sorting
GAC: glutaminase C
GFP: green fluorescent protein
GLS: glutaminase
KO: knockout
MeCP2: methyl CpG binding protein 2
mTOR: mammalian target of rapamycin
PAMAM: poly(amidoamine)
RTT: Rett syndrome
WT: wild type
