# Supplementary material for: Dendrimer-conjugated glutaminase inhibitor selectively targets microglial glutaminase in a mouse model of Rett syndrome
Source: Theranostics. 2020 Apr 27;10(13):5736–48. doi: 10.7150/thno.41714 (PMC7254984; doi:10.7150/thno.41714)
Supplement: Supplementary file 1 — Supplementary figures and information. [file thnov10p5736s1.pdf]

SUPPLEMENTARY INFORMATION

Dendrimer-conjugated glutaminase inhibitor selectively targets microglial glutaminase in a mouse model of Rett syndrome

Elizabeth Smith Khoury[1,2] , Anjali Sharma[2], Rajsekhar R Ramireddy[2], Ajit G.Thomas[3], Jesse Alt[3], Amanda Fowler[1], Rana Rais[3], Takashi Tsukamoto[3,4], Mary E. Blue[4,5], Barbara Slusher[3,4], Sujatha Kannan*[1,4,5], Rangaramanujam M. Kannan *[2,4,6,7]

[1]Department of Anesthesiology and Critical Care Medicine, Johns Hopkins University School of Medicine, Baltimore MD, 21205

[2]Center for Nanomedicine, Department of Ophthalmology, Wilmer Eye Institute Johns Hopkins University School of Medicine, Baltimore MD, 21231

[3]Johns Hopkins Drug Discovery, Johns Hopkins University School of Medicine, Baltimore MD, 21205

[4]Department of Neurology, Johns Hopkins University School of Medicine, Baltimore MD, 21205

[5]Hugo W. Moser Research Institute at Kennedy-Krieger Inc, Baltimore MD, 21205

[6]Kennedy Krieger Institute – Johns Hopkins University for Cerebral Palsy Research Excellence, Baltimore, MD 21287

[7]Departments of Chemical and Biomolecular Engineering, and Materials Science and Engineering, Johns Hopkins University, Baltimore MD, 21218

*Corresponding authors:

Rangaramanujam M. Kannan

Professor of Ophthalmology, Center for Nanomedicine at the Wilmer Eye Institute

400 North Broadway, Baltimore, Maryland 21231, USA

Tel.: +1 443-287-8634; Fax: +1 443-287-8635; e-mail: krangar1@jhmi.edu

Sujatha Kannan, MD

Mailing address: Department of Anesthesiology and Critical Care Medicine,

Charlotte Bloomberg Children's Center 6318D,

1800 Orleans Street Baltimore, MD, 21287

Tel: 410- 955-6412

E-mail: skannan3@jhmi.edu

1. Compound characterization:

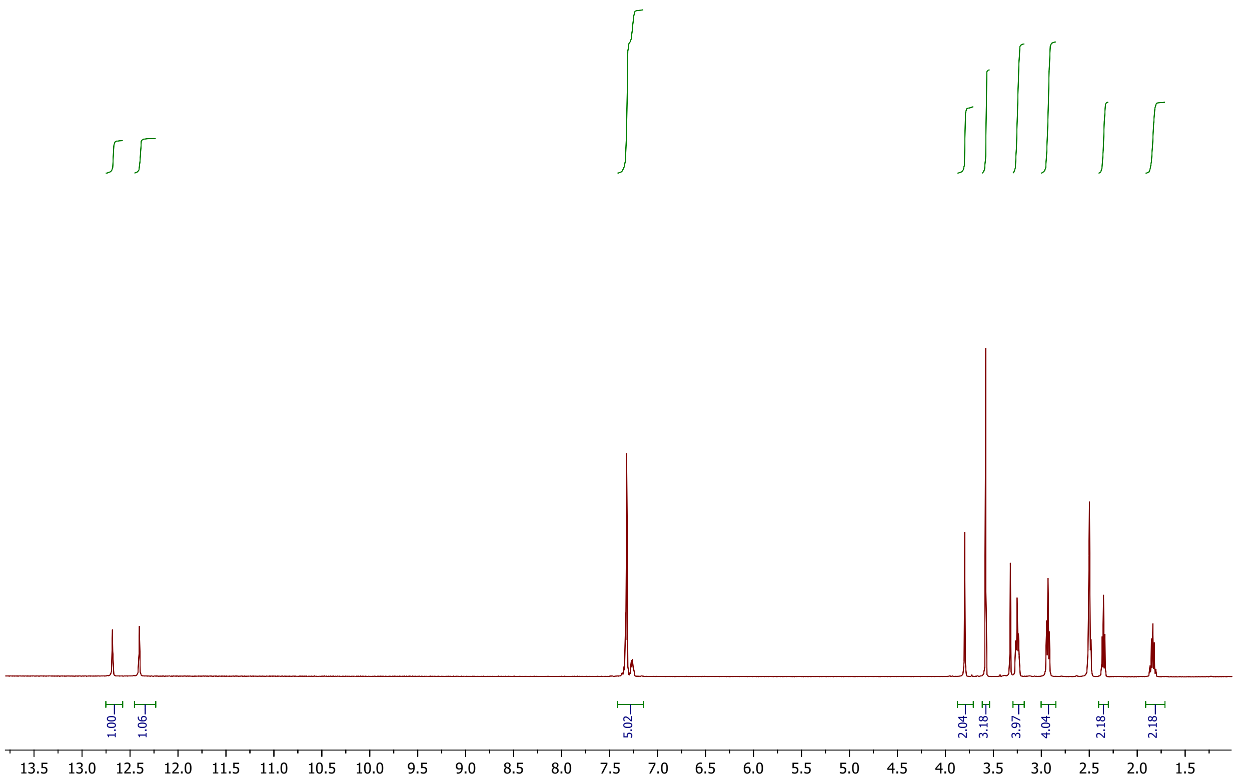

Figure S1. ^{1}H NMR of compound **3**.

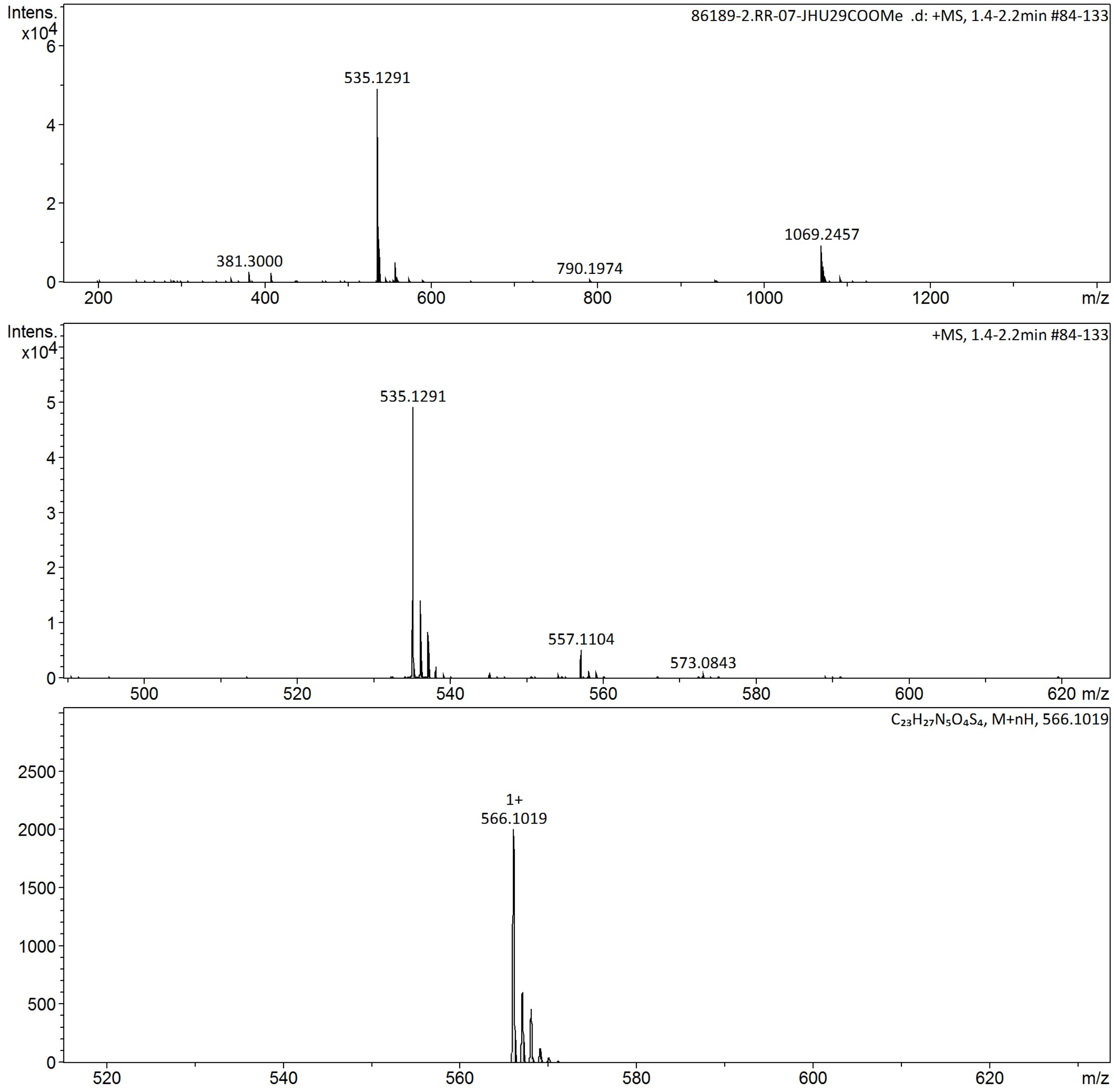

Figure S2. ESI-MS spectrum of compound **3**.

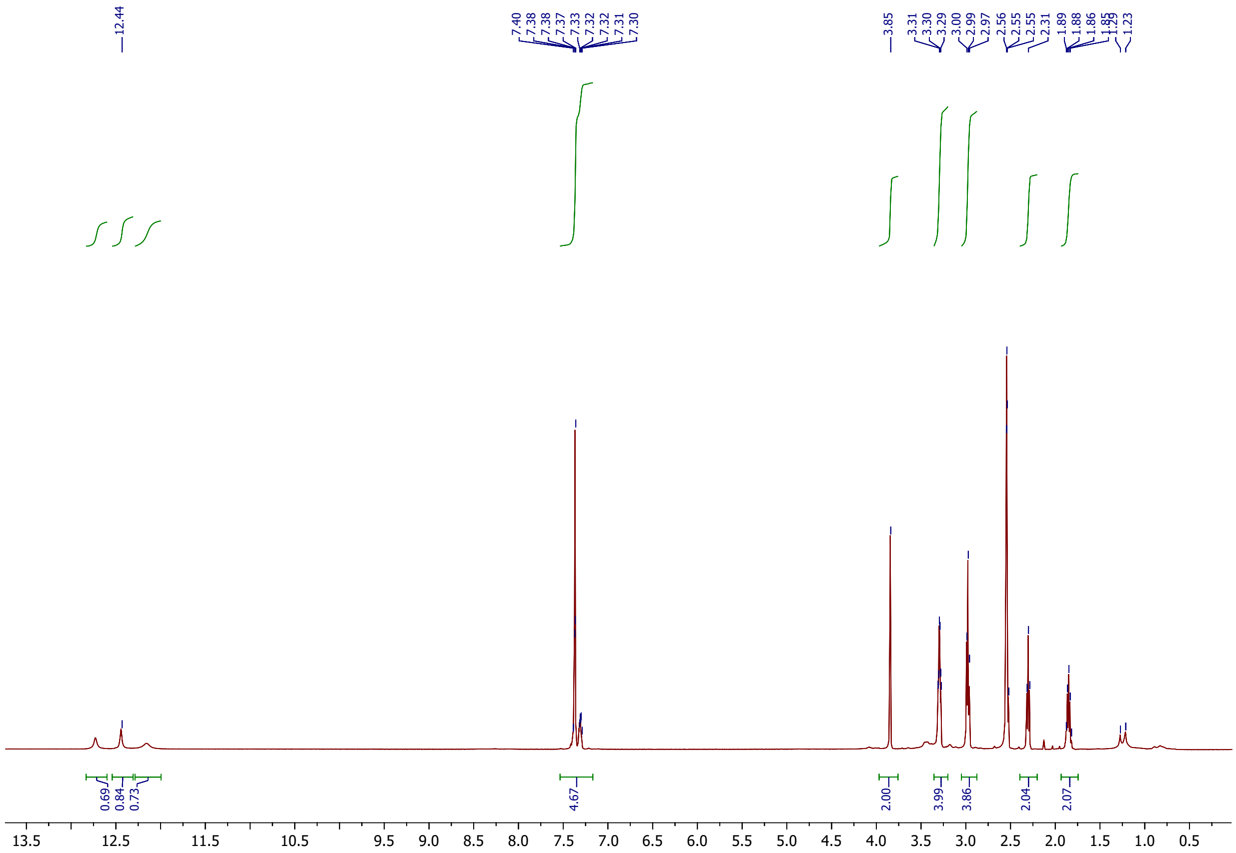

Figure S3. ^{1}H NMR of compound **4**.

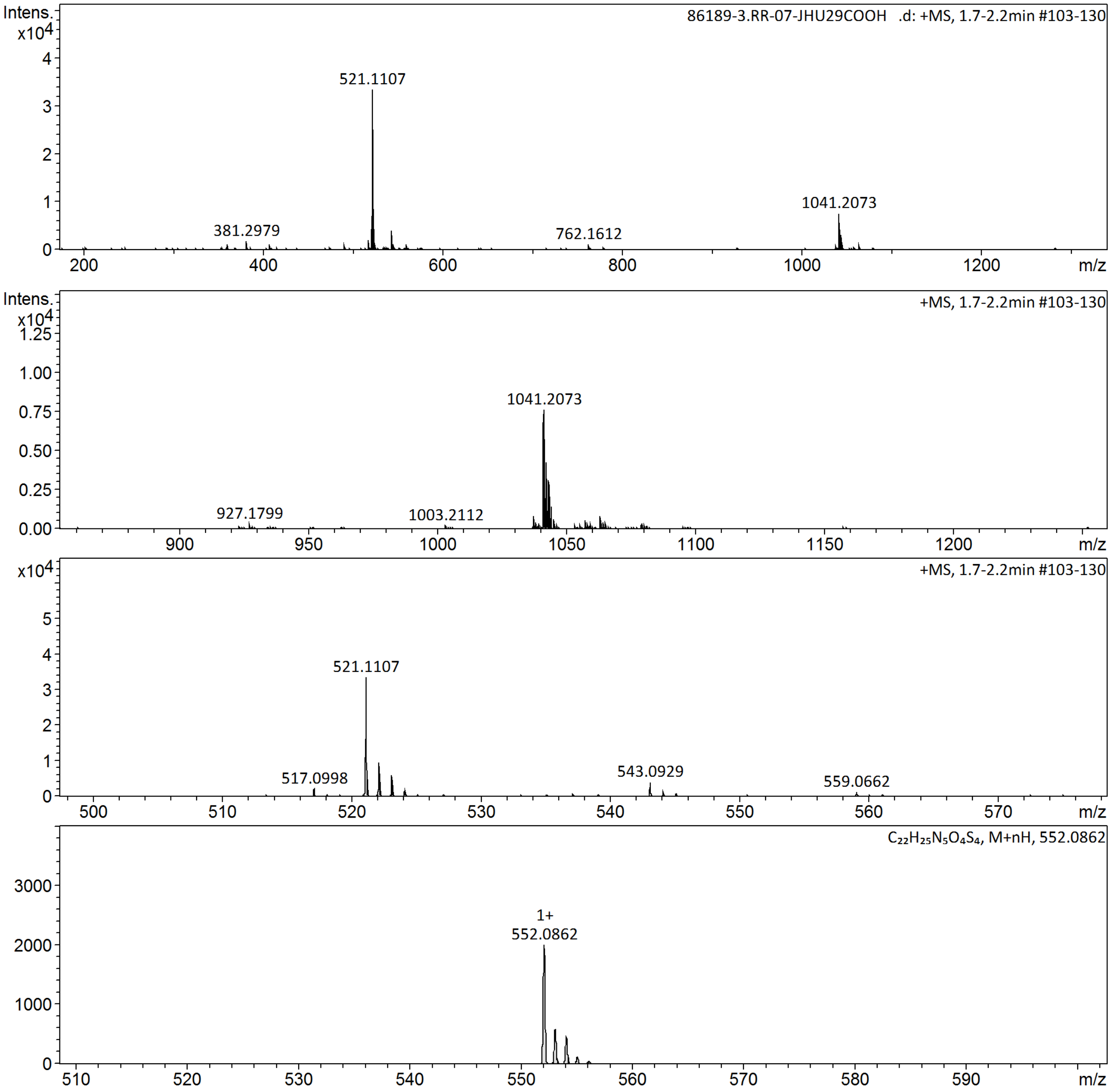

Figure S4. ESI-MS spectrum of compound **4**.

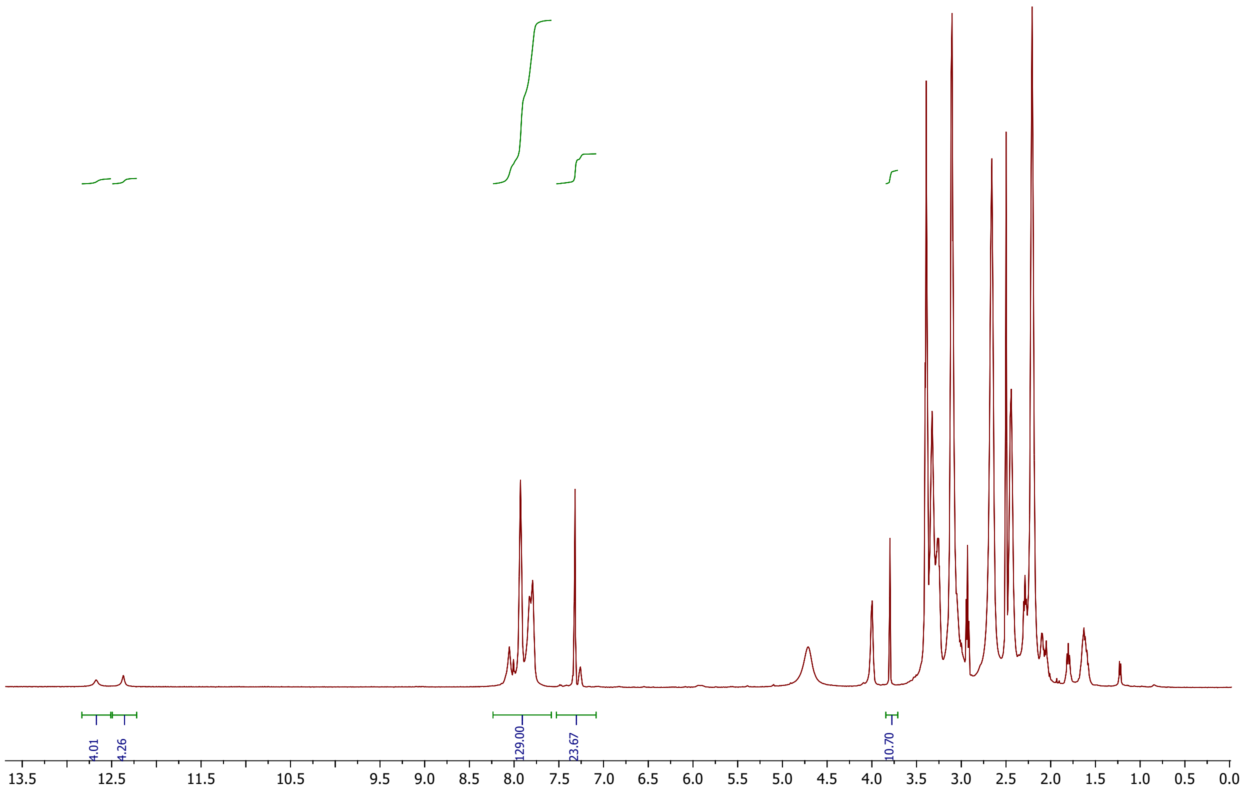

Figure S5. ^{1}H NMR of compound **6 (D-JHU29)**.

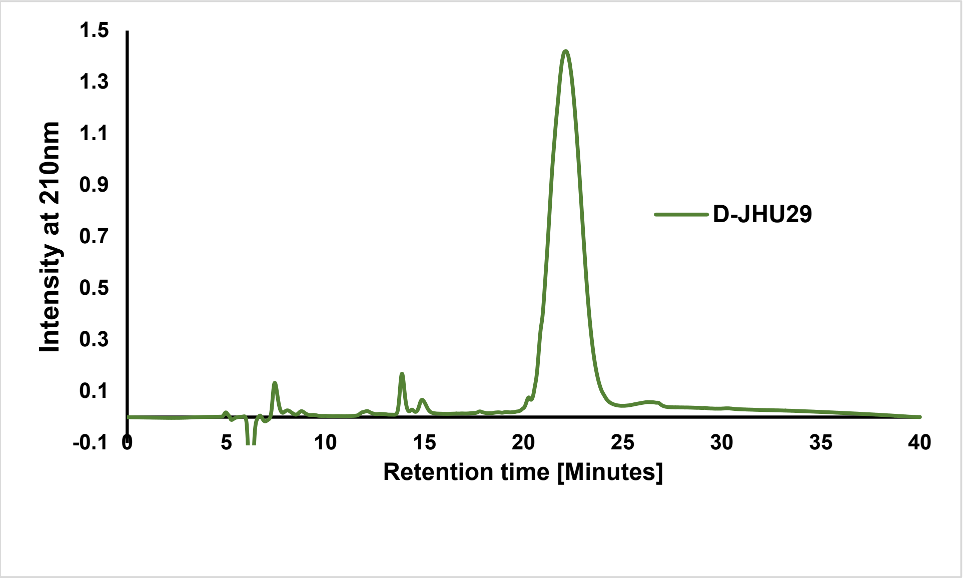

Figure S6. HPLC chromatogram of compound **6** (D-JHU29).

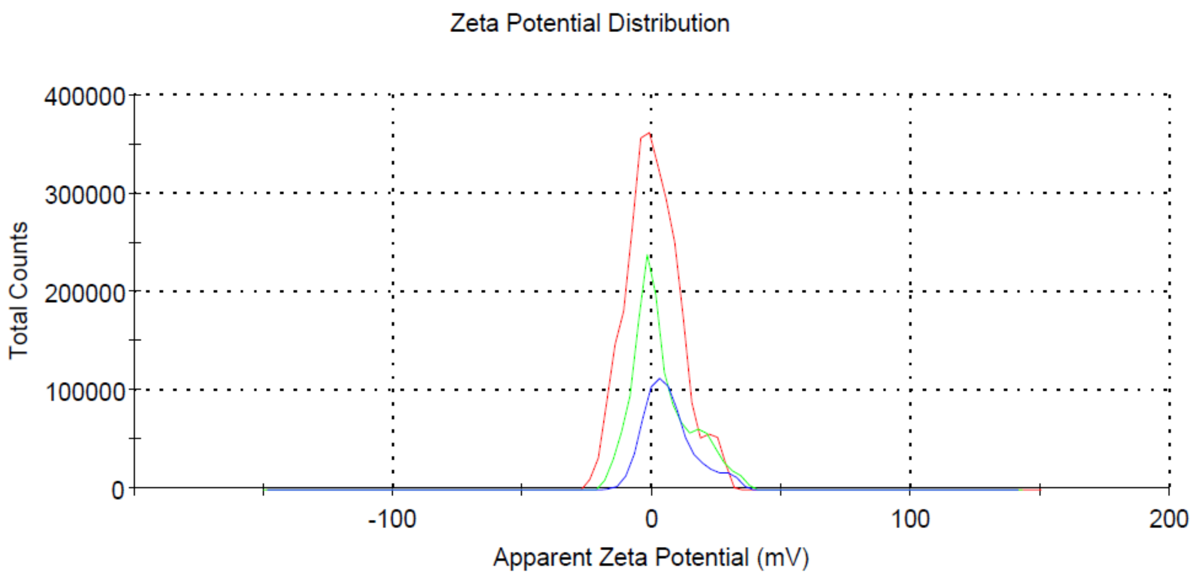

Figure S7. The zeta potential distribution of D-JHU29.

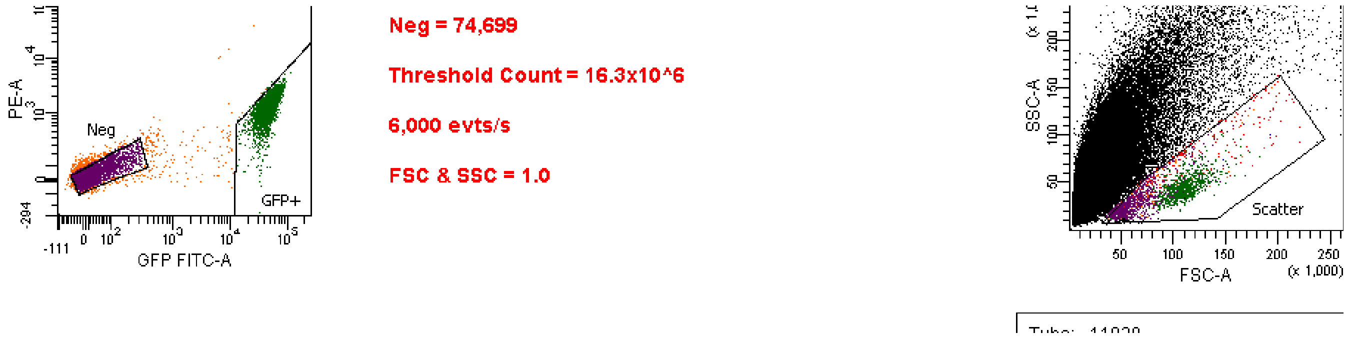

Figure S8. Flow cytometry gating scheme. Approximately 8.0 x10^4 GFP+ and GFP- cells were collected per sample. Events were processed at a rate of 6,000 events/s and with a threshold count of 16.3 x 10^6.

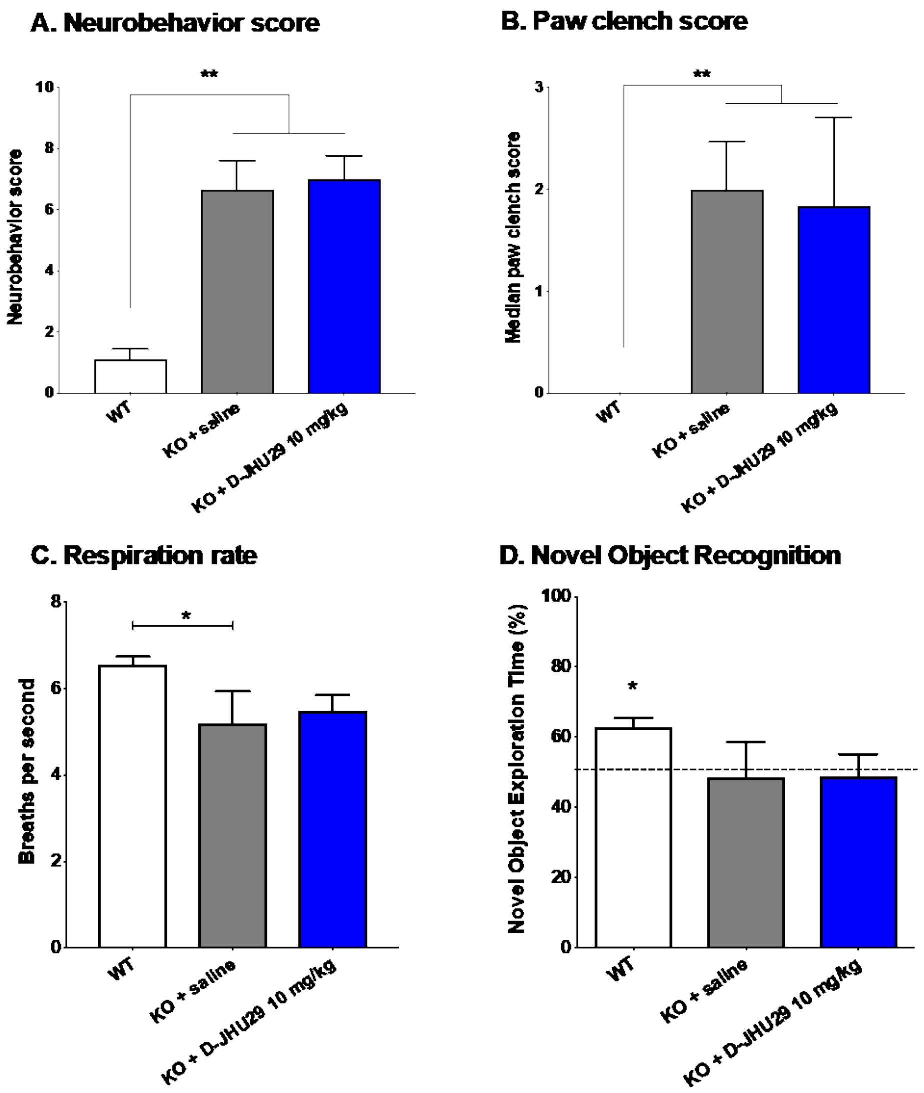

Figure S9. Non-motor phenotypic assessments in *Mecp2*-KO mice after twice weekly D-JHU29 treatment. A. Neurobehavioral assessment is significantly greater in *Mecp2*-KO mice indicating significant phenotypic changes in these mice and there is no significant improvement with treatment. **B.** The predominant neurobehavior subscore, paw clench also shows no improvement change with D-JHU29 treatment. **C.** Respiration rate is significantly decreased in *Mecp2*-KO mice (gray bars) but is not recovered with D-JHU29 administration (blue bars). **D.** No improvements in novel object recognition were observed at D-JHU29 treatment. Only wild type (WT) mice (white bars) show on average more time spent with the novel object than what would be expected at chance (50%). *$p < 0.05$, ** $p < 0.01$

Neurobehavior Rubric:

Neurobehavioral phenotype was scored using the following measures by a blinded observer. All subscores were added to create an overall/composite neurobehavior score.

Subscore:

<u>Mobility</u>

- 0 normal, active
- 1 decreased spontaneous movement
- 2 no spontaneous movement, only moves when prodded
- 3 no movement

<u>Gait</u>

- 0 walks normally
- 1 waddling
- 2 waddling, walks on toes (paws not flat on ground)
- 3 waddling, feet displaced outward, paws clenched when walking

<u>Tremors</u>

- 0 none
- 1 sporadic
- 2 intermittent
- 3 continuous

<u>Paw Clenching</u>

- 0 none
- 1 1-2 paws
- 2 3 paws or both fore limbs/hind limbs
- 3 all paws

<u>Clench time</u>

- 0 none
- 1 sporadic
- 2 intermittent (patterned)
- 3 continuous

<u>Paw wringing</u>

- 0 none
- 1 some front paw
- 2 some front and back paw
- 3 continuous

<u>Respiration</u>

- 0 regular
- 1 irregular
- 2 too fast or too slow
- 3 apnic

<u>Composite Score:</u> addition of all sub-scores
